# Do the various leisure forms have equal effects on mental health? A longitudinal analysis of self-selected leisure activities

**DOI:** 10.3389/fpubh.2023.1134854

**Published:** 2023-05-16

**Authors:** Junyi Bian, Zubing Xiang

**Affiliations:** ^1^School of Physical Education, Guangzhou Sport University, Guangzhou, China; ^2^Department of Human Performance and Health Education, Western Michigan University, Kalamazoo, MI, United States; ^3^School of Physical Education, Chongqing University, Chongqing, China

**Keywords:** leisure, classification, self, assessment, psychology

## Abstract

The deteriorating trends of unbalanced income, progressive age-related health problems, and loss of traditional ties necessitate ever-flexible interventions that are helpful to overcome a decline in Chinese adults' mental health. This study aimed to test whether engagement in different domains of leisure is associated with a composite index of mental health, both concurrently and subsequently. Longitudinal data including 10,968 participants (females = 5,804) with a mean age of 46.01 years in the Chinese General Social Survey (CGSS) were analyzed using generalized estimating equations with a logit link. The results showed that face-to-face experiences, such as sports with all ORs of < 1 at the significance level of α = 0.01 and meeting with all ORs of < 1 at the significance level of α = 0.01 except daily in-person meeting frequency, are important for protecting mental health owing to the increasing social support by building close ties. The results also indicate that online leisure with all ORs < 1 at the significance level of α = 0.01 has positive effects on lowering the odds of depression. In addition, receptive cognitive leisure, such as watching television or movies with all ORs < 1 at the significance level of α = 0.05 level, was not consistently associated with depression. However, active cognitive leisure, such as reading with all ORs of < 1 at a significance level of α = 0.01, was associated with lower odds of depression.

## 1. Introduction

Currently, China suffers from collective mental health decline. Based on the data from the Chinese Mental Health Report (1), the percentage of adults satisfied with their mental health was 31.4%, while the percentage of those dissatisfied with their mental health was 45.9%. Furthermore, the report also indicated that the total mental health condition of the population has decreased compared with that 10 years ago. Despite this, mental distress or depression is commonly undiagnosed and underestimated and is often undertreated (2). Distress causes functional impairments in daily life and is associated with increased risks of cardiovascular disease, dementia, diabetes, stroke, and both specific and unspecific mortality (3–6). Against these backgrounds, identifying ways to reduce or prevent mental health concerns is particularly important. Furthermore, the deteriorating trends of unbalanced income, progressive age-related health problems, and loss of traditional ties necessitate ever-flexible interventions that are increasingly related to maintaining or achieving a healthy lifestyle (7).

In China, participating in cultural activities and organized travel trips are popular leisure trends that may help reduce depression (8). However, these activities require physical, social, creative, and imaginative participation from the participants. Although previous studies provide strong evidence that engaging in cultural activities (such as going to galleries and museums) and organized travel is associated with lower rates of depression (9, 10), organized travel may not be easily or equally accessible to all as it may be subject to higher socioeconomic barriers unlike other activities, like reading, watching television, or meeting with relatives or friends. Furthermore, cultural activities depend on the contextual environment of a person's life, and inequalities in access may likely increase with aging. It raises the question of whether a wide range of self-selected and participatory leisure activities that can be done at home, many of which are inexpensive or free, contribute to effectively preventing depression. Leisure activity is one of the known determinants of health and wellbeing. Previous studies have compellingly indicated that exercise as a form of leisure is consistently identified as having antidepressant effects in terms of physiological and neurological functions (11, 12). However, the effects of other flexible leisure activities such as reading, watching television, sewing, and digital leisure in protecting mental health have, to date, received less attention.

The current study investigated how engaging in different forms of leisure, such as exercise, reading, sewing, watching television or movies at home, meeting friends, and digital leisure, are associated with mental health protection. Considering social ties as a well-known element in protecting mental health in the real world, one possible mechanism of how digital leisure connects with mental health is that the interpersonal virtual networks allow for the exchange of social resources, which generates a sense of control and competence, thereby creating beliefs of resilience that make stress more bearable (13, 14). These perceptions of virtual social ties are described as “buffers” against life stress, especially when life stress is high. In addition, choosing reading and watching television/movies may be alternative elements in protecting mental health because cognitive stimulation, one possible mechanism connecting leisure and mental health, improves symptoms of depression and anxiety (15). However, the mechanism behind those is still unclear, and understanding the role of leisure is an important topic that merits further exploration.

Thus, choosing certain leisure forms should imply having greater opportunities to activate social ties and maintain the levels of social ties that may be beneficial for mental health, as leisure can become a source of happiness and personal growth (16) and detachment from stressful activities (17). Therefore, this study (1) tested the concurrent and subsequent associations between different leisure forms and a composite index of mental health; (2) discerned which leisure activities, including exercise, reading, sewing, watching television or movies at home, meeting friends, and digital leisure, help the most to protect mental health; and (3) discussed the possible anti-depressive effects of specific leisure activities that seem particularly important post-pandemic, partially after witnessing the exceptional consequences of the global COVID-19 pandemic: social distancing and whole-city lockdowns.

### 1.1. Literature review and hypothesis development

#### 1.1.1. Online and offline leisure and mental health: a social capital perspective

Previous research has stressed that leisure creates different contexts that can facilitate the development of companionship, friendship, and social support, thus reducing feelings of loneliness and protecting people from psychological distress (18, 19). While leisure provides many interactive contexts that can be beneficial for mental health, studies involving issues of social capital and support suggest that many of these processes occur in settings where leisure is experienced through in-person scenarios (1, 20, 21).

The social capital theory is an umbrella term that broadly refers to the resources embedded in social ties that can be accessed through individual or collective social connections (22). Processes of social capital circulation are, thus, key to activating those kinds of resources that have been conceptualized as social capital since the studies by Akdere (23). From this perspective, Spini et al. (24) stressed the importance of being actively engaged in social connections to establish forms of social support that can provide protection against mental problems. In-person social interactions fostered feelings of reciprocity and help to circulate those kinds of social resources embedded in the social ties (25), e.g., emotional and material aids provided by strong social ties. In addition, China's high-speed economic development has induced large changes in society. For instance, some traditional social ties have been lost, meaning that residents are more likely to live alone. Against this background, some individuals spend their time engaging in sedentary and domestic activities, and Alkahtani argued (26) that they have fewer opportunities to actively foster social ties, thus losing their capacity to cultivate and circulate social capital. On the one hand, existing research suggests that traditional forms of leisure involving face-to-face social ties can provide enough opportunities to create a process of social capital circulation that helps to protect mental health (25). On the other hand, online communication and digital leisure vary in style but are becoming indispensable, especially due to the pandemic, and have provided many different opportunities for participants to connect through sporting apps, outdoor adventure comments, and sharing cultural activities.

Although digital leisure has been gaining popularity, the question of whether social capital is activated by virtual social ties remains unclear, as do the relationships between digital leisure and the odds of depression. It is easy to understand that the digital world can facilitate peer communication beyond time and space. Nakagomi et al.'s (27) findings supported the role of online communication with friends in preventing clinical depression among young people. In addition, online communication could be particularly useful among older adults as, today, families are often geographically dispersed. Bano (28) attributed the positive effects of virtual interactions with others to affect wellbeing via a decreased sense of loneliness as well as increased social engagement, which are core elements of activating social resources. Although Gardiner (29) described in his study that many requests for help in and through virtual interactions often go unanswered, prevention of social capital circulation is decreasing due to flourishing online-specific communities, such as running groups on Facebook or sharing tourism tips on Twitter. Furthermore, some studies have demonstrated that increases in virtual engagement are correlated with fewer in-person social connections, but the positive effects of digital leisure on preventing mental health should be not ignored. Thus, it is reasonable to develop a digital leisure hypothesis.

H1: Increasing in-person exercise participation is associated with a lower probability of mental depression.

H2: Increased participation in meetings with friends is associated with a lower probability of mental depression.

H3: Increased participation in digital leisure is associated with a lower probability of mental depression.

### 1.2. Leisure and mental health: cognitive behavioral perspective

Although leisure involving social interactions was most consistently associated with lower odds of depression, there were still associations between participating in hobbies, baking, and cooking and depression, suggesting that these activities involve another active ingredient of protecting mental health, although they do not necessarily result in social connections (29, 30). Based on a cognitive behavioral therapy perspective, effective stimulation has three critical components: collaborative construction, verbal or visible intervention, and reframing (31). Each of these components involves a simulated objective in the participant's mind, which means that the aforementioned leisure activities such as hobbies, baking, or cooking actually do not involve interacting with peers virtually or in person but instead create simulated interactions to protect mental health.

In this aspect, there has been a growing body of research that demonstrates the effects of engagement in cultural activities on reducing depression. These cultural activities are commonly split into those with active engagement, such as singing, dancing, or doing artistic activities, and those with receptive engagement, involving art that has been created and then experienced by an audience (10). Cognitive stimulation, corresponding to those forms of active and receptive cultural activities, is not ignored, although there are many physical and social components involved. Previous studies have indicated that cultural engagement (such as going to galleries and art museums) is associated with lower odds of depression (9, 32), but these activities may not be accessible to all. Participating in cultural activities may be subject to more socioeconomic barriers than other leisure activities, and inequalities in access may increase with age (33). On the contrary, due to their cognitive stimulation and similarity to cultural activities, at-home activities like reading, watching television, and sewing, which are affordable or free, can help prevent depression. Specifically, considering that there is a difference between active and receptive cognitive stimulations, developing these cognitive stimulation spheres of leisure is reasonable.

H4: Higher rates of active reading are significantly associated with a lower probability of mental depression.

H5: Higher rates of receptive TV or movie watching at home have negative effects on protecting mental health.

H6: Higher rates of sewing are associated with a lower probability of mental depression.

## 2. Materials and methods

### 2.1. Sample

Participants were drawn from the Chinese General Social Survey (CGSS), which is a nationally representative study of 10,968 individuals with a mean age of 46.01 years in China. The initial cohort was first investigated in 2011 and followed up every 2 years. We used public data from CGSS waves with which engagement in leisure activities was consistently measured (2013–2017). Those three waves of investigation datasets were treated as longitudinal investigations in at least two respects. First, this national survey sample is replenished with younger cohorts every 8 years, which means those three waves of samples should be not replenished. Second, each wave survey is strongly representative of the whole population due to strict probability sampling. Specifically, each wave investigation considered its representativeness from two aspects: economic factors as a vertical axis and geographic units as a horizontal axis. For instance, while 2,801 counties were treated as primary sampling basic units, five different sampling frames were employed depending on the economic factors such as Gross Domestic Product (GDP) and human development index (detailed information shown in Table 1). Ultimately, 125 first sampling units were conducted by simple random sampling, and the ratio between urban and rural blocks was 295:205 in the second sampling unit and the ratio of people living in urban and rural areas was 5,900:4,100, which satisfies the sampling design. Each sampling unit had a confidential interval of 95% and a sampling error of no more than 3%. The least sample size was 5,335. However, considering the issues of invalid response and response rate, the sample size of each wave was set at 10,000. Restricting the sample in the current study to participants with complete data on depression and leisure activity participation in three waves produced a final sample of 10,968 participants and 32,904 observations.

**Table 1:** A summary of sampling strategy.

**Sampling-frame**	**First unit**	**Second unit**	**Third unit**	**Ultimate unit**
Frame-1	15	60	120	1,200
Beijing	5	5 × 4 = 20	20 × 2 = 40	40 × 10 = 400
Tianjin	5	5 × 4 = 20	20 × 2 = 40	40 × 10 = 400
Shanghai	5	5 × 4 = 20	20 × 2 = 40	40 × 10 = 400
Frame-2	16	64	128	1,280
Eastern	5	5 × 4 = 20	20 × 2 = 40	40 × 10 = 400
Central	6	6 × 4 = 24	24 × 2 = 48	48 × 10 = 480
Western	5	5 × 4 = 20	20 × 2 = 40	40 × 10 = 400
Frame-3	30	30 × 4 = 120	120 × 2 = 240	240 × 10 = 2,320
Frame-4	42	42 × 4 = 168	168 × 2 = 336	336 × 10 = 3,360
Frame-5	22	22 × 4 = 88	88 × 2 = 176	176 × 10 = 1,760
Total	125	500	1,000	10,000

### 2.2. Measurement

#### 2.2.1. Outcome

Depression was measured at every wave with a 3-item self-evaluated depression scale (SEDS), as specified in Table 2. This self-reported measurement can identify people at risk of developing depression. The total score ranges from 1 to 5, with lower scores indicating more severe symptoms. The SEDS had good internal consistency across waves (Cronbach's alpha ranged from 0.71 to 0.85). We recoded a cutoff value of three or fewer to indicate the presence of depression (34).

**Table 2:** Standardized factor loadings, composite reliability coefficients, and average variance extracted for the self-evaluated depression scale.

**Construct/items**	β			**CR**			**AVE**		
	**2013**	**2015**	**2017**	**2013**	**2015**	**2017**	**2013**	**2015**	**2017**
Item-1	0.849^**^	0.718^**^	0.727^**^	0.878	0.726	0.775	0.707	0.642	0.654
Item-2	0.894^**^	0.762^**^	0.703^**^						
Item-3	0.776^**^	0.567^**^	0.563^**^						

N = 10,968, β, standardized factor loadings; CR, composite reliability coefficients; AVE, average variance extracted.

** p < 0.01.

Item-1: How do you rate your mental wellbeing? Item-2: In the past 4 weeks, how often have the mental problems interfered with your work or other daily activities? Item-3: In the past 4 weeks, how often have you felt depressed?

#### 2.2.2. Independent variables

We selected six items from this CGSS questionnaire that were measured consistently from 2013 to 2017, including questions on a wide range of leisure activities. Participants were asked how often they (1) read books, magazines, or newspapers (*Reading*); (2) attend meetings with friends or relatives, community, or other interest groups (*Meeting*); (3) watch TV or movies at home (*Watching*); (4) play a sport or attend an exercise club (*Exercising*); (5) carry out sewing, knitting, or embroidery (*Sewing*); and (6) browse the internet for leisure (*Internet*). We collapsed the responses into four categories, representing daily engagement (once a day/several times daily), weekly engagement (once a week/several times a week), monthly engagement (once a month/several times a month), and yearly or no engagement (never/not in the last half year, and once a year/several times a year).

#### 2.2.3. Moderated effects of demographic characteristics

We included a range of demographic and socioeconomic-related confounders. Demographic confounders were age in years and gender (men vs. women). The socioeconomic confounders were educational attainment (none, elementary school, middle and high school, vocational school, undergraduate degree, graduate degree, and above).

### 2.3. Statistical analyses

We first described the sociodemographic characteristics of the sample at baseline. We then investigated whether engagement in leisure activities was associated with concurrent and subsequent depression. Concurrent models included simultaneous leisure activity engagement and depression measurement, with estimates averaged across all waves (2013–2017).

Longitudinal models tested the association between activity engagement in one wave (2013–2017) and depression in the subsequent wave (2013–2017). Thus, the longitudinal model estimated the associations between leisure activity engagement and change in depression after 4 years. We fitted population-averaged panel data models using generalized estimating equations (GEE) with the logit links. This allowed us to include repeated measures, with waves clustered within an individual. We used an exchangeable correlation matrix to optimize model power and efficiency, although GEE is relatively robust to the choice of correlation structure (35). We modeled depression with a binomial distribution, logit link, and robust standard errors. All models were presented before and after adjustment for confounders. Each leisure activity was treated as a continuous variable ranging from daily to monthly to indicate whether there was overall evidence for the moderation effect of the demographic variables on the odds of depression. The missing data on exposures and confounders were imputed using multiple imputations. We used logistic regression according to variable type, generating 20 imputed data sets using all variables included in the analyses. The analyses were performed using R 4.3 and SAS 9.4.

## 3. Results

In total, 10,968 participants provided data on leisure activities and depression in the three waves of CGSS. More than half of the participants (52.92%) were women, 11.30% did not receive any education, and 73.36% received medium education. The mean age of the participants was 46.01 years. Overall, 16.08% of the sample size met the diagnostic criteria for depression. The prevalence of depression differed according to several socioeconomic and health-related factors, as did the frequency of engagement in leisure activities (Table 3). Specifically, the frequency of engagement differed considerably across leisure activities; moreover, 58.28% of participants participated in Watching weekly, whereas <10% of participants participated in Sewing. After Watching, the most common categories of activity were Internet and Meeting (Table 4).

**Table 3:** A summary of the sample at the baseline and percentage of the sample with depression at the baseline according to demographic characteristics.

	**Overall**	**Depression**	
		**No**	**Yes**
Overall		9,204 (83.92%)	1,764 (16.08%)
**Gender**			
Male	5,164 (47.08%)	4,333 (83.91%)	831 (19.09%)
Female	5,804 (52.92%)	4,871 (83.92%)	933 (16.07%)
**Education**			
None	1,239 (11.30%)	1,038 (83.78%)	201 (16.23%)
Elementary school	2,386 (21.75%)	2,003 (83.95%)	383 (16.05%)
Middle and high school	5,031 (45.87%)	4,200 (83.48%)	831 (16.52%)
Vocational school	63 (5.74%)	51 (80.95%)	12 (19.05%)
College and university	2,066 (18.84%)	1,756 (85.00%)	310 (15.00%)
Graduate and above	183 (1.67%)	156 (85.25%)	27 (14.75%)
Age	46.01 (16.84)		

**Table 4:** Frequency distribution of leisure activities at baseline.

**Frequency**	**Reading**	**Meeting**	**Watching**	**Exercising**	**Sewing**	**Internet**
Daily	1,245 (11.35%)	324 (2.95%)	6,392 (58.28%)	1,998 (18.22%)	202 (1.84%)	4,848 (44.20%)
Weekly	1,189 (10.84%)	1,253 (11.42%)	2,565 (23.39%)	1,926 (17.56%)	333 (3.04%)	906 (8.26%)
Monthly	1,272 (11.60%)	2,926 (26.67%)	818 (7.46%)	906 (8.26%)	527 (4.80%)	242 (2.21%)
None	7,261 (66.21%)	6,464 (59.37%)	1,192(10.87%)	6,137(55.96%)	9,905 (90.35%)	4,971 (45.33%)

### 3.1. Concurrent association

Before adjusting for confounders, we found evidence that more frequent activities, such as Reading, Meeting, Internet, and Exercising were associated with lower odds of depression (Table 5). In contrast, there was no evidence that more frequent Sewing was associated with decreased odds of depression. After adjustment for confounders, there was also no evidence that more frequent Sewing was associated with lower odds of depression. Compared to participants who did not spend time exercising, those exercising weekly had 39% [OR = 0.61, 95% CI (0.50, 0.71)] lower odds of depression, which was very similar to those who reported browsing the internet weekly [OR = 0.57, 95% CI (0.44, 0.70)]. In addition, engagement in exercise showed more of a dose–response relationship with lower odds of depression. Daily, weekly, and monthly participation in sport were respectively associated with a 43% [OR = 0.57, 95% CI (0.49, 0.67)], 39% [OR = 0.61, 95% CI (0.50, 0.71)], and 21% [OR = 0.79, 95% CI (0.60, 0.98)] decrease in the odds of depression when compared to not participating in sports or exercising.

**Table 5:** Concurrent models testing the associations between frequency of engagement in leisure activities and the odds of depression.

	**Model 1: Unadjusted**			**Model 2: Adjusted**		
	**OR**	**95%CI**	***p*** **-value**	**OR**	**95%CI**	***p*** **-value**
**Watching**						
Daily	1.01	0.83–1.18	0.93	0.94	0.77–1.11	0.49
Weekly	0.92	0.74–1.10	0.42	0.94	0.74–1.13	0.53
Monthly	0.87	0.63–1.10	0.30	0.87	0.62–1.12	0.36
**Reading**						
Daily	0.87	0.71–1.03	0.14	**0.80**	**0.64–0.96**	**0.02**
Weekly	**0.80**	**0.63–0.97**	**0.04**	0.85	0.67–1.04	0.15
Monthly	**0.71**	**0.56–0.86**	**< 0.01**	**0.79**	**0.62–0.95**	**0.03**
**Meeting**						
Daily	1.07	0.77–1.37	0.61	0.98	0.70–1.25	0.88
Weekly	**0.71**	**0.58–0.85**	**< 0.01**	**0.77**	**0.62–0.92**	**< 0.01**
Monthly	**0.59**	**0.50–0.68**	**< 0.01**	**0.68**	**0.57–0.78**	**< 0.01**
**Exercising**						
Daily	**0.64**	**0.54–0.73**	**< 0.01**	**0.57**	**0.49–0.67**	**< 0.01**
Weekly	**0.57**	**0.47–0.67**	**< 0.01**	**0.61**	**0.50–0.71**	**< 0.01**
Monthly	**0.72**	**0.55–0.89**	**< 0.01**	**0.79**	**0.60–0.98**	**< 0.01**
**Sewing**						
Daily	1.12	0.70–1.53	0.56	1.11	0.69–1.52	0.59
Weekly	0.95	0.64–1.24	0.73	0.92	0.62–1.21	0.60
Monthly	1.02	0.75–1.28	0.88	1.03	0.75–1.30	0.87
**Internet**						
Daily	**0.22**	**0.19–0.24**	**< 0.01**	**0.52**	**0.43–0.61**	**< 0.01**
Weekly	**0.34**	**0.26–0.40**	**< 0.01**	**0.57**	**0.44–0.70**	**< 0.01**
Monthly	**0.32**	**0.19–0.44**	**< 0.01**	**0.51**	**0.29–0.71**	**< 0.01**

N = 10,968. For all leisure activities, no engagement was the reference category. Model 2 was adjusted for gender, education, and age. The bold text indicates a p-value of < 0.05.

### 3.2. Longitudinal association

Before adjusting for confounders, we found evidence that indicated that a higher frequency of Reading, Meeting, Internet, and Exercising was associated with lower odds of depression (Table 6), which was the same with the concurrent model. However, in the fully adjusted model, it was evidenced that daily reading was associated with subsequent lower odds of depression. Participants who read daily had 20% [OR = 0.80, 95% CI (0.64, 0.96)] lower odds of depression 4 years later and participants who met with friends or organized activities weekly had 23% [OR = 0.77, 95% CI (0.62, 0.92)] lower odds of subsequent depression. Both weekly and monthly sports and Exercising were associated with a 39% and 21% [weekly 95% CI (0.60, 0.98) and monthly 95% CI (0.5, 0.7)] reduction in the odds of depression 4 years later, respectively. The effects of higher rates of Internet were associated with lower odds of depression 4 years later. Finally, reading weekly was not statistically associated with lower odds of subsequent depression [OR = 0.85, 95% CI (0.67, 1.04)].

**Table 6:** Longitudinal models testing associations between the frequency of engagement in leisure activities and the odds of depression in the subsequent waves (4 years later).

	**Model 1: Unadjusted**			**Model 2: Adjusted**		
	**OR**	**95%CI**	***p*** **-value**	**OR**	**95%CI**	***p*** **-value**
**Watching**						
Daily	1.01	0.83–1.18	0.93	0.94	0.77–1.11	0.49
Weekly	0.92	0.74–1.10	0.40	0.94	0.74–1.13	0.53
Monthly	0.87	0.63–1.10	0.28	0.87	0.62–1.12	0.36
**Reading**						
Daily	0.87	0.71–1.03	0.14	**0.80**	**0.64–0.96**	**0.02**
Weekly	**0.80**	**0.63–0.97**	**0.04**	0.85	0.67–1.04	0.15
Monthly	**0.71**	**0.56–0.86**	**< 0.01**	**0.79**	**0.62–0.95**	**0.03**
**Meeting**						
Daily	1.07	0.77–1.37	0.61	0.98	0.70–1.25	0.88
Weekly	**0.72**	**0.58–0.85**	**< 0.01**	**0.77**	**0.62–0.92**	**< 0.01**
Monthly	**0.59**	**0.50–0.68**	**< 0.01**	**0.68**	**0.57–0.78**	**< 0.01**
**Exercising**						
Daily	**0.64**	**0.54–0.73**	**< 0.01**	**0.57**	**0.49–0.67**	**< 0.01**
Weekly	**0.57**	**0.47–0.67**	**< 0.01**	**0.61**	**0.50–0.71**	**< 0.01**
Monthly	**0.72**	**0.55–0.89**	**< 0.01**	**0.79**	**0.60–0.98**	**< 0.01**
**Sewing**						
Daily	1.12	0.70–1.53	0.58	1.11	0.69–1.52	0.59
Weekly	0.95	0.64–1.24	0.73	0.92	0.62–1.21	0.60
Monthly	1.02	0.75–1.28	0.88	1.02	0.75–1.30	0.87
**Internet**						
Daily	**0.22**	**0.19–0.24**	**< 0.01**	**0.52**	**0.43–0.61**	**< 0.01**
Weekly	**0.34**	**0.26–0.40**	**< 0.01**	**0.57**	**0.44–0.70**	**< 0.01**
Monthly	**0.32**	**0.19–0.44**	**< 0.01**	**0.51**	**0.29–0.71**	**< 0.01**

N = 10,968. For all leisure activities, no engagement was the reference category. Model 2 was adjusted for gender, education, and age. The bold text indicates a p-value of < 0.05.

### 3.3. Moderated effects of confounders on association

Before and after adjusting for confounders, we found evidence of interactions between Watching and gender, on the one hand, and of lower odds of depression, on the other hand (Table 7). Men had more than 15% [OR = 0.85, 95% CI (0.72, 0.96)] and 18% [OR = 0.82, 95% CI (0.70, 0.95)] lower odds of depression concurrently and sequentially 4 years later, respectively.

**Table 7:** Models testing whether the association between the frequency of leisure activities and the odds of depression differs according to gender.

	**Concurrent (*****n*** = **10,968)**						**Longitudinal (*****n*** = **10,968)**					
	**Model 1: Unadjusted**			**Model 2: Adjusted**			**Model 1: Unadjusted**			**Model 2: Adjusted**		
	**OR**	**95% CI**	***p***	**OR**	**95% CI**	***p***	**OR**	**95% CI**	***p***	**OR**	**95% CI**	***p***
Watching	**0.85**	**0.72–0.96**	**0.02**	**0.82**	**0.70–0.95**	**0.01**	**0.85**	**0.72–0.97**	**0.01**	**0.82**	**0.70–0.95**	**0.01**
Reading	0.90	0.79–1.01	0.08	0.94	0.82–1.06	0.34	0.90	0.79–1.01	0.08	0.94	0.83–1.16	0.34
Meeting	1.06	0.96–1.15	0.24	1.05	0.95–1.15	0.34	1.06	0.96–1.16	0.24	1.05	0.95–1.14	0.33
Exercising	1.00	0.87–1.12	0.99	0.97	0.84–1.09	0.64	1.00	0.87–1.13	0.99	0.97	0.85–1.10	0.64
Sewing	0.93	0.77–1.09	0.41	0.93	0.77–1.09	0.40	0.93	0.77–1.09	0.43	0.93	0.77–1.09	0.40
Internet	1.06	0.86–1.26	0.54	1.09	0.91–1.28	0.29	1.06	0.85–1.26	0.61	1.09	0.91–1.28	0.29

Interaction terms are reported (gender * activity), the female group was the reference category, with each activity treated as continuous to indicate whether there was overall evidence for an interaction. The bold text indicates a p-value of < 0.05.

Adjusting for age as a confounder, the more the older adults engaged with the internet, the higher their odds of depression, based on the concurrent and longitudinal models [OR= 1.01, 95% CI (1.00, 1.02)]. Longitudinally, there was one more piece of evidence showing that the association between Meeting and depression differed according to age group, but this was not present in the concurrent model (Table 8). For those who were older, meeting with friends and relatives was associated with 1% lower odds of subsequent depression [OR = 0.99, 95% CI (1.00, 1.01)].

**Table 8:** Models testing whether the association between the frequency of leisure activities and the odds of depression differs according to age.

	**Concurrent (*****n*** = **10,968)**						**Longitudinal (*****n*** = **10,968)**					
	**Model 1: Unadjusted**			**Model 2: Adjusted**			**Model 1: Unadjusted**			**Model 2: Adjusted**		
	**OR**	**95% CI**	***p***	**OR**	**95% CI**	***p***	**OR**	**95% CI**	***p***	**OR**	**95% CI**	***p***
Watching	0.99	0.99–1.00	0.97	0.99	1.00–1.01	0.804	1.00	0.99–1.01	0.969	0.99	0.99–1.00	0.809
Reading	0.99	0.99–1.00	0.12	0.99	1.00–1.01	0.224	0.99	0.99–1.00	0.117	0.99	0.99–1.00	0.196
Meeting	1.00	1.00–1.01	0.01	1.00	1.00–1.01	0.032	**0.99**	**1.00–1.01**	**0.002**	**0.99**	**1.00–1.01**	**0.025**
Exercising	0.99	0.99–1.00	0.37	0.99	0.99–1.00	0.229	1.00	0.99–1.00	0.370	0.99	0.99–1.00	0.231
Sewing	0.99	0.99–1.00	0.43	0.99	0.99–1.00	0.461	1.00	0.99–1.00	0.411	0.99	0.99–1.00	0.453
Internet	**1.01**	**1.00–1.02**	**< 0.01**	**1.01**	**1.01–1.03**	**< 0.01**	**1.01**	**1.00–1.02**	**0.001**	**1.01**	**1.01–1.02**	**< 0.001**

Interaction terms are reported (age * activity), with each activity treated as continuous to indicate whether there was overall evidence for an interaction. The bold text indicates a p-value of < 0.05.

Similarly, for educational levels (Table 9), we found evidence that the higher the participants' education levels, the higher the odds of depression, when looking at the category “Internet” [OR = 1.06, 95% CI (1.01, 1.10)], from the unadjusted model. In contrast, this evidence is not present in the adjusted and longitudinal models.

**Table 9:** Models testing whether the association between the frequency of leisure activities and the odds of depression differs according to education.

	**Concurrent (*****n*** = **10,968)**						**Longitudinal (*****n*** = **10,968)**					
	**Model 1: Unadjusted**			**Model 2: Adjusted**			**Model 1: Unadjusted**			**Model 2: Adjusted**		
	**OR**	**95% CI**	***p***	**OR**	**95% CI**	***p***	**OR**	**95% CI**	***p***	**OR**	**95% CI**	***p***
Watching	1.00	0.98–1.03	0.61	1.01	0.98–1.03	0.37	1.00	0.98–1.03	0.60	0.99	0.99–1.00	0.37
Reading	1.01	0.99–1.03	0.25	1.01	0.99–1.03	0.26	1.01	0.99–1.03	0.26	0.99	0.99–1.00	0.27
Meeting	0.99	0.97–1.00	0.17	0.99	0.97–1.01	0.70	0.98	0.97–1.01	0.20	0.99	1.00–1.01	0.71
Exercising	0.99	0.97–1.02	0.70	0.99	0.97–1.02	0.94	1.00	0.97–1.01	0.72	0.99	0.99–1.00	0.95
Sewing	1.02	0.98–1.04	0.21	1.02	0.98–1.04	0.26	1.01	0.98–1.04	0.23	0.99	0.99–1.00	0.28
Internet	**1.06**	**1.01–1.10**	**0.01**	1.03	0.98–1.07	0.15	**1.06**	**1.00–1.10**	**0.04**	1.01	1.00–1.02	0.20

Interaction terms are reported (education * activity), with each activity treated as continuous to indicate whether there was overall evidence for an interaction. The bold text indicates a p-value of < 0.05.

## 4. Discussion

We explored the associations between engagement in a wide range of leisure activities and a composite index of mental health in a large nationally representative cohort of adults in China, concurrently and consequently. The study aimed at identifying which types of leisure domains were associated with psychological distress and the possible mechanism behind them. Leisure is well-known for creating a context for social interaction, and it is vital to further investigate whether there is a clear distinction between online and offline scenarios when dealing with the rise in the popularity of online leisure (36, 37), while looking at whether the required social capital is activated equally both online and offline and exploring how different leisure domains can provide benefits to mental health through exercise, cognitive stimulations, and virtual social ties. Such questions are attracting more attention due to online leisure becoming indispensable, especially during a global pandemic.

As expected in the face-to-face and online leisure hypotheses (H1, H2, and H3), participating in sport daily, weekly, or monthly was consistently associated with lower odds of concurrent and subsequent depression (H1). In addition, engagement in sports showed more of a dose–response relationship with lower odds of depression. Meeting with friends or relatives weekly or monthly was consistently associated with lower odds of concurrent and subsequent depression (H2), but there was no evidence to support the association between meeting daily and depression. Moreover, leisure activities that involve social connections are particularly beneficial for protecting mental health (38, 39). Similarly, we have found that both participating in sports or attending fitness clubs and meeting friends or relatives were most strongly associated with lower odds of depression. Interestingly, experiencing leisure through browsing the internet was also strongly associated with lower odds of depression (H3). To understand these virtual interaction benefits, we can posit that virtual interactions through the internet provided feelings of support and care from virtual networks, which may involve the causal mechanism of how social capital is activated (25, 40). For example, one active benefit of the internet is the provision of social resources that can reduce loneliness (41), which helped answer the research hypothesis (H3), i.e., whether online leisure should be associated with a lower probability of mental depression due to activating social capital.

Another objective of the current study was to investigate whether leisure activities involving different types of cognitive stimulations—active vs. receptive—provide different benefits in terms of preventing depression from the cognitive therapy perspective. The results indicated that reading weekly was associated with lower odds of depression, but only concurrently and not longitudinally (H4). There was no evidence to support that sewing was associated with concurrent and sequent depression (H6), although the reason may be related to the fact that sewing is not popular in China (participatory frequency of < 10%). However, we did not only find strong evidence, for example, of the relationship between exercise and depression, but we found strong evidence that the higher the frequency of reading activities, the lower the odds of depression. Monthly reading activities were associated with lower odds of depression concurrently and subsequently, which was consistent with previous research. Leisure activities involving cognitive elements have been shown to have additional effects in reducing the risk of depression in adults (42) and the more obvious effects of cognitive interventions than traditional social engagement for older adults experiencing depression (43). Although it was widely accepted that the primary reasons for Exercising and Meeting in reducing the odds of depression were related to social interactions (44), there were still associations between Reading and depression. Although reading activities do not involve social interactions, the process of reading produces cognitive and imaginative simulations, which is a key element of treatments for depression, such as cognitive behavioral therapy (CBT), and is effective in reducing depressive symptoms (45). In addition, the relationship between Reading and depression was not a direct dose–response relationship, which needs further investigation in the future. In summary, leisure involving cognitive elements should not be limited to reading. Leisure activities containing imaginative and creative elements should be extended into daily life due to the evidence that reading activities provide positive benefits for reducing the odds of depression. In contrast, watching television or movies was not consistently associated with lower odds of depression (H5), which may be related to its sedentary and passive nature (46–48).

When considering the effects of confounders, the effect of Exercising was independent of a range of confounders. However, Watching (e.g., TV, movie at home), Meeting (friends and relatives), and Internet were not consistently associated with depression with the influence of confounders. Specifically, older adults that were sensitive to meeting with friends or relatives were associated with lower odds of depression. More frequent Internet was only associated with higher odds of subsequent depression in those with higher educational backgrounds. Men had more than 15% lower odds of depression than women concurrently [OR = 0.85, 95% CI (0.72, 0.96)], and subsequently 4 years later for Watching, which may be explained by a large proportion of housework taken by women at home traditionally in China. For confounders, we found no evidence that engagement in leisure activities differentially affected depression in men and women except for Watching, which may be related to large gender differences in the frequency of this category. We found age differences in the association between Meeting and depression. For those who were older, the frequency of meeting with friends and relatives was higher, which was associated with lower odds of subsequent depression. Similarly, the higher the educational level of participants, the higher the odds of depression.

## 5. Strengths and limitations

One of the strengths of the current study was the use of a large nationally representative cohort of adults, which allowed us to investigate population-averaged concurrent and longitudinal associations between leisure activities and depression while controlling for a range of confounders. However, the way in which questions were asked in CGSS limited the investigation of previous factors that influence depression. In addition, one limitation of the measurement of engagement was that leisure activities were measured by frequency, without considering other dimensions such as artistic creativity and exercise intensity. Future research should focus on the measurement of different types of creativity, which clearly explain the causality between activity engagement and depression.

## 6. Conclusion

Our findings indicate that engagement in a wide range of leisure activities is associated with lower odds of depression for adults. Although we found no evidence that a higher frequency of reading activities is associated with lower odds of depression, the results indicated that leisure activities involving active cognitive stimulations provide additional active benefits for reducing depression compared with receptive stimulations. Alternatively, online leisure is another source of social resources that can be helpful in overcoming depression. Given the protective nature of easily accessible activities in lowering depression, policymakers should consider how adults can be supported to engage in leisure activities involving exercise, cognitive stimulations, and virtual social ties. While the current study results have contributed to the existing body of social capital and cognitive behavior theory in the mechanism of mental health protection, an egocentric network behind depression should be not ignored. The core point behind the different modes of leisure mobilizes social resources, and a simulated objective is created and activated at first, giving up an egocentric idea, which will help to further determine which online leisure activities beneficially cultivate participation in communities or group activities that occur in real interactions. Although the potential of online leisure has been evident in our maintenance of ties with peers virtually, close in-person interaction seems to be irreplaceable, and further research on social capital and cognitive behaviors is needed to advance our understanding of how social capital is circulated between the real and digital worlds of leisure and its links with mental health.

## Data availability statement

The original contributions presented in the study are included in the article/supplementary material, further inquiries can be directed to the corresponding author.

## Author contributions

JB: conceptualization, methodology, software, validation, formal analysis, investigation, resources, data curation, and writing—original draft. ZX: conceptualization, methodology, validation, investigation, resources, writing—review and editing, project administration, and funding acquisition. Both authors contributed to the article and approved the submitted version.
